# 2806. The Mechanism of β-Lactam Antibiotic Action: Structural Analysis of *Mycobacterium abscessus* l,d-Transpeptidases, d,d-Carboxypeptidase and penicillin-binding-proteins binding by imipenem, ceftaroline, and amoxicillin

**DOI:** 10.1093/ofid/ofad500.2417

**Published:** 2023-11-27

**Authors:** Eunjeong Shin, Mary Nantongo, Khalid M Dousa, Christopher R Bethel, Magdalena A Taracila, Barry N Kreiswirth, Steven M Holland, Robert A Bonomo

**Affiliations:** Case Western Reserve University, Cleveland, Ohio; Case Western Reserve University, Cleveland, Ohio; Louis Stokes Cleveland Department of Veterans Affairs Medical Center, Cleveland, Cleveland, Ohio; Louis Stokes Cleveland Department of Veterans Affairs Medical Center, Cleveland, Cleveland, Ohio; Case Western Reserve University, Cleveland, Ohio; Center for Discovery and Innovation, Hakensack Meridian Health, Nutley, New Jersey; National Institutes of Health, Bethesda, Maryland; Louis Stokes Cleveland Department of Veterans Affairs Medical Center, Cleveland, Cleveland, Ohio

## Abstract

**Background:**

*M. abscessus* (Mab) is among the most clinically challenging pathogens. There are significant gaps remaining in mechanism of β-lactams action in *Mab*. β-Lactams preferentially inactivate one or multiple different L,D-Transpeptidases (LDTs) and penicillin-binding proteins (PBPs). Founded on our LDT binding data and in vitro synergistic efficacy of imipenem (IPM), ceftaroline (CFT), and amoxicillin (AMX) in Mab, this study aimed to identify binding affinities, conformational changes and the thermal stability of five LDTs and PBPs via β-lactam binding for further structure-activity relationship.

**Methods:**

Binding affinity was measured by kinetic studies, MS analysis, or Bocillin-FL inhibition assay. We investigated changes in the secondary structure of five LDTs (LDT1-5), d,d-carboxypeptidase, PBPB, and PBP-lipo in response to bind of IPM, CFT, and AMX using circular dichroism (CD) spectroscopy in the far-UV region. To assess the thermal stability of these LDTs, we employed a Differential Scanning Fluorimetry (DSF) assay.

**Results:**

CD analysis revealed a significant negative shift of LDT1, LDT2, and PBPB upon binding with either single or combination of imipenem (IPM) and ceftriaxone (CFT), particularly in the 210-220 nm range. Using DSF, IPM and CFT binding to LDTs, DDC and PBPB were found to alter the thermal stability of these protein enzymes. IPM induced a decrease in the melting temperature (*T_m_*) in the range of 2.6-6.8 ˚C for LDTs and PBPB, while caused an increase in Tm for DDC. CFT caused decrease in Tm in the range of 1.4-3 ˚C for LDTs but increase in Tm for DDC and PBPB. In contrast, amoxicillin (AMX) only targeted DDC, PBPB, and PBP-lipo and caused changes in Tm. The magnitude of the change in Tm varied between combinations and single β-lactams. Although there was no evidence of a correlation between changes in protein stability upon ligand binding and ligand binding affinity, IPM with high binding affinity (lower value of *K_i,app_*) resulted in larger decreases in Tm compared to CFT.

**Figure:**
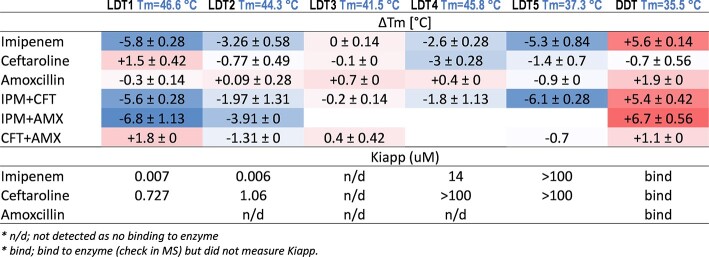

**Conclusion:**

β-Lactam binding in single and combination leads to distinct structural changes of LDTs and PBPs, likely reflecting synergistic interactions between the two β-lactams. Further crystallography will be required to gain a better understanding of the induced structural changes.

**Disclosures:**

**Robert A. bonomo, MD**, Entasis, Merck, VenatoRx, Wockhardt: Grant/Research Support

